# Case for diagnosis. Eyelid edema and erythematous papules disseminated on the face^⋆^^⋆⋆^

**DOI:** 10.1016/j.abd.2019.11.016

**Published:** 2020-08-16

**Authors:** Ana Cristina M. Garcia, Ângela Marques Barbosa, Marilda Aparecida Milanez Morgado de Abreu, Carlos Zelandi Filho

**Affiliations:** a Dermatology Service, Hospital Regional de Presidente Prudente, Universidade do Oeste Paulista, Presidente Prudente, SP, Brazil; b Department of Health Postgraduate Programs, Instituto de Assistência Médica ao Servidor Público Estadual, São Paulo, SP, Brazil; c Private Laboratory, Presidente Prudente, SP, Brazil

**Keywords:** Clinical diagnosis, Granulomatous disease, chronic, Skin abnormalities

## Abstract

Lupus miliaris disseminatus faciei or acne agminata is a chronic inflammatory disorder of the skin, considered an intriguing entity due to its pathogenesis, which is still largely speculative. It has been linked to tuberculosis, sarcoidosis, rosacea, and other granulomatous diseases, but it is considered an independent entity.

## Case report

The authors report the case of a 47-year-old female patient who presented asymptomatic erythematous papules on the central region of the face for eight months, in addition to periocular edema. She initially associated them with the use of a hair dye, but the lesions persisted despite interrupting its use. The lesions did not worsen with sun exposure or any other factor. The patient reported no associated symptoms, comorbidities, or continuous use of medications.

**Figure 1:**
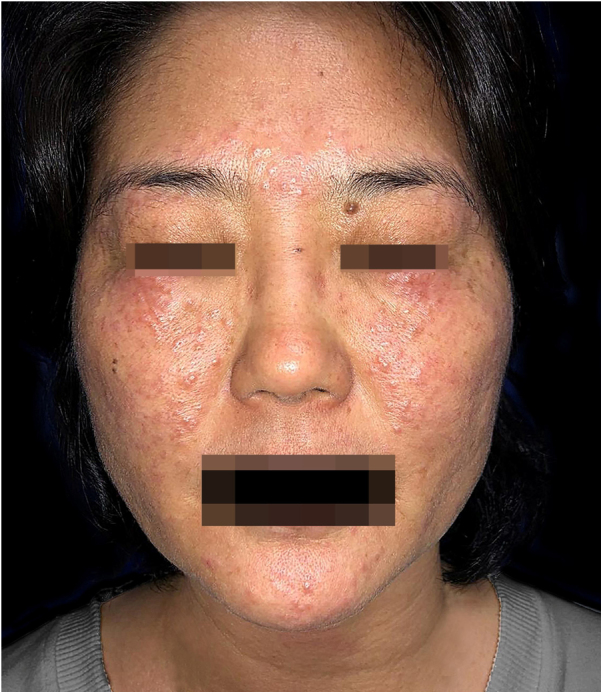
Erythematous papules on the malar areas, forehead and chin. Plaques formed by coalescence of papules on both eyelids.

At dermatological examination, multiple erythematous papules were observed, measuring from 1 to 3 mm, distributed over the malar area, forehead and chin. Over the eyelids, these papules coalesced to form plaques, and there was mild bilateral edema (Fig. 1). The skin around the papules presented no changes. No changes were observed in other organs and systems.

**Figure 2:**
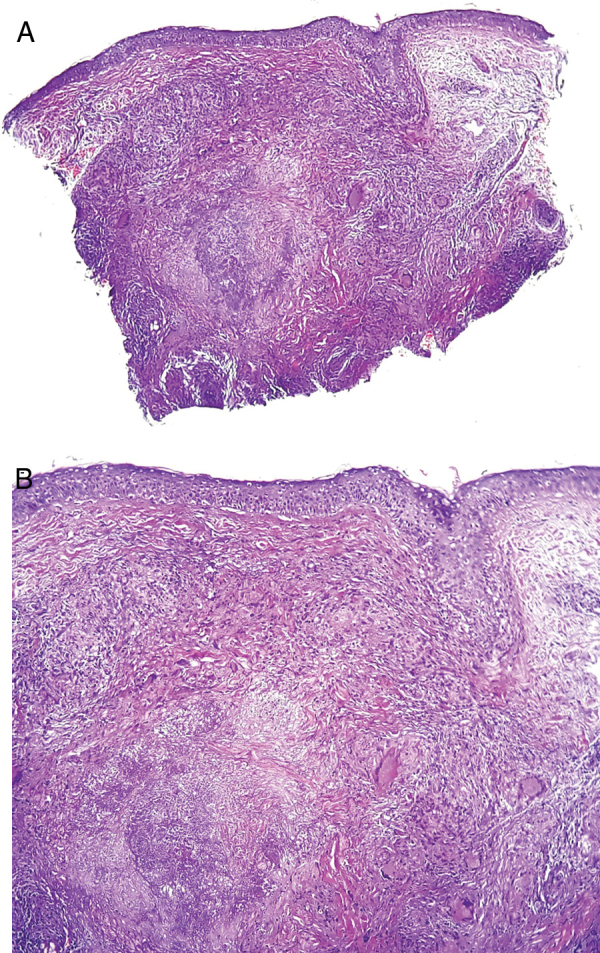
(A) No major changes in the epidermis. Granulomatous inflammation in the middle and deep dermis. Caseous necrosis in the center of the granulomatous process (Hematoxylin & eosin, ×40). (B) Focal necrosis and granulomatous inflammation with giant cells (Hematoxylin & eosin, ×100). Special stains were negative for acid-fast bacilli and fungi.

The patient had been previously submitted to treatment with prednisone 40 mg/day for four months, without response. An extensive laboratory investigation (Table 1) was performed, as well as biopsy of a lesion (Fig. 2A and B).

**Table 1:** Complementary exams – investigation panel.

Serology for HIV, hepatitis B, hepatitis C, and VDRL
PTH, calcium, phosphorus, 25-hydroxyvitamin D, copper, zinc, sodium, potassium
Cell blood count, iron, ferritin, glucose, triglycerides
Renal function (urea and creatinine)
Thyroid function (TSH, fT4), ANA
Liver function (transaminases, bilirubins, alkaline phosphatase)
Protein electrophoresis
Stool parasitology (3 samples)
PPD
CD4, CD8, CD3
Chest X-ray

Note: All exams were within normal range.

After histopathological examination of the lesion and other complementary exams, treatment with minocycline and topical betamethasone was then proposed.  

## What is your diagnosis?

a) Granulomatous rosacea
b) Sarcoidosis
c) Acne agminata
d) Cutaneous tuberculosis

## Discussion

Described by Radcliffe-Crocker in 1903, lupus miliaris disseminatus faciei (LMDF) or acne agminata is a rare chronic granulomatous inflammatory disease of the face, of unknown etiology. It mainly affects adolescents and young adults of both sexes.^1^, ^2^, ^3^

It is characterized by asymptomatic papules and nodules, brownish or yellowish in color, located mainly on the central region of the face, typically on and around the eyelids. Extrafacial involvement can occur. The onset is abrupt, and the condition usually regresses spontaneously in 12–24 months, leaving punctiform and atrophic scars.^1^, ^2^, ^3^

Histologically, it is characterized by epithelioid granulomas similar to tuberculosis, sarcoidosis, granulomatous rosacea, tuberculoid leprosy, or other granulomatous diseases. Its pattern may vary according to the stage of the lesion: early lesions are characterized by nonspecific and non-granulomatous inflammation, whereas well-developed lesions may present epithelioid granulomas with central necrosis, without central necrosis (sarcoid granuloma), or with abcess formation.^4^, ^5^

Its etiopathogenesis remains unknown. It was believed to be a manifestation of cutaneous tuberculosis; however, this theory is no longer accepted, as the presence of *M. tuberculosis* has not been demonstrated, in addition to the fact that the condition does not improve with anti-tuberculosis therapy.^1^, ^2^, ^6^ Many authors considered it to be a variant of granulomatous rosacea, a hypothesis that has also been discarded, as in rosacea there is a greater predilection for the malar region, association with diffuse erythema, telangiectasias, exacerbation with corticosteroids, and absence of scarring after resolution.^1^, ^3^ It can also resemble sarcoidosis, but this usually presents systemic manifestations, as well as laboratory and imaging alterations, not observed in LMDF.^1^, ^2^, ^3^, ^6^

Due to the lack of knowledge of the etiopathogenesis, its treatment is challenging; many drugs have been used as possible treatments, including dapsone, clofazimine, minocycline, doxycycline, tetracycline, prednisone, and isotretinoin.^2^, ^7^, ^8^

Although there are no well-established criteria for the diagnosis of LMDF, the authors reached the diagnosis of acne agminata due to the distribution of the lesions and clinical characteristics suggestive of granulomatous disease of the face, and by excluding other differential diagnoses after biopsy and complementary exams.

## Financial support

None declared.

## Authors’ contributions

Ana Cristina M Garcia: Approval of the final version of the manuscript; elaboration and writing of the manuscript; intellectual participation in propaedeutic and/or therapeutic conduct of studied cases; critical review of the literature; critical review of the manuscript.

Ângela Marques Barbosa: Approval of the final version of the manuscript; intellectual participation in propaedeutic and/or therapeutic conduct of studied cases.

Marilda Aparecida Milanez Morgado de Abreu: Intellectual participation in propaedeutic and/or therapeutic conduct of studied cases; critical review of the manuscript.

Carlos Zelandi Filho: Intellectual participation in propaedeutic and/or therapeutic conduct of studied cases.

## Conflicts of interest

None declared.
